# Supplementary material for: Coumarin Derivatives Inhibit ADP-Induced Platelet Activation and Aggregation
Source: Molecules. 2022 Jun 23;27(13):4054. doi: 10.3390/molecules27134054 (PMC9268609; doi:10.3390/molecules27134054)
Supplement: Supplementary file 1 [file molecules-27-04054-s001.zip › molecules-1743677-supplementary.pdf]

molecules

MDPI

Article

Coumarin Derivatives Inhibit ADP-Induced Platelet Activation and Aggregation

Ping-Hsun Lu [1,2,†], Tzu-Hsien Liao [1,†], Yau-Hung Chen [3], Yeng-Ling Hsu [4], Chan-Yen Kuo [5], Chuan-Chi Chan [6], Lu-Kai Wang [7], Ching-Yuh Chern [4,*] and Fu-Ming Tsai [5,*]

[1] Department of Chinese Medicine, Taipei Tzu Chi Hospital, Buddhist Tzu Chi Medical Foundation, New Taipei City 231, Taiwan; pinghsunlu@gmail.com (P.-H.L.); a0958651263@gmail.com (T.-H.L.)
[2] School of Post-Baccalaureate Chinese Medicine, Tzu Chi University, Hualien 970, Taiwan
[3] Department of Chemistry, Tamkang University, New Taipei City 251, Taiwan; yauhung@mail.tku.edu.tw
[4] Department of Applied Chemistry, National Chia-Yi University, Chiayi City 600, Taiwan; s1100243@gmail.ncyu.edu.tw
[5] Department of Research, Taipei Tzu Chi Hospital, Buddhist Tzu Chi Medical Foundation, New Taipei City 231, Taiwan; cykuo863135@gmail.com
[6] Department of Laboratory Medicine, Taipei Tzu Chi Hospital, Buddhist Tzu Chi Medical Foundation, New Taipei City 231, Taiwan; kiki1205@tzuchi.com.tw
[7] Department of Life Sciences, Ministry of Science and Technology, Taipei 106, Taiwan; keratin14kaikai@gmail.com
* Correspondence: cychern@mail.ncyu.edu.tw (C.-Y.C.); afu2215@gmail.com (F.-M.T.); Tel.: +886-5-2717970 (C.-Y.C.); +886-2-66289779-5793 (F.-M.T.)
† These authors contributed equally to this work.

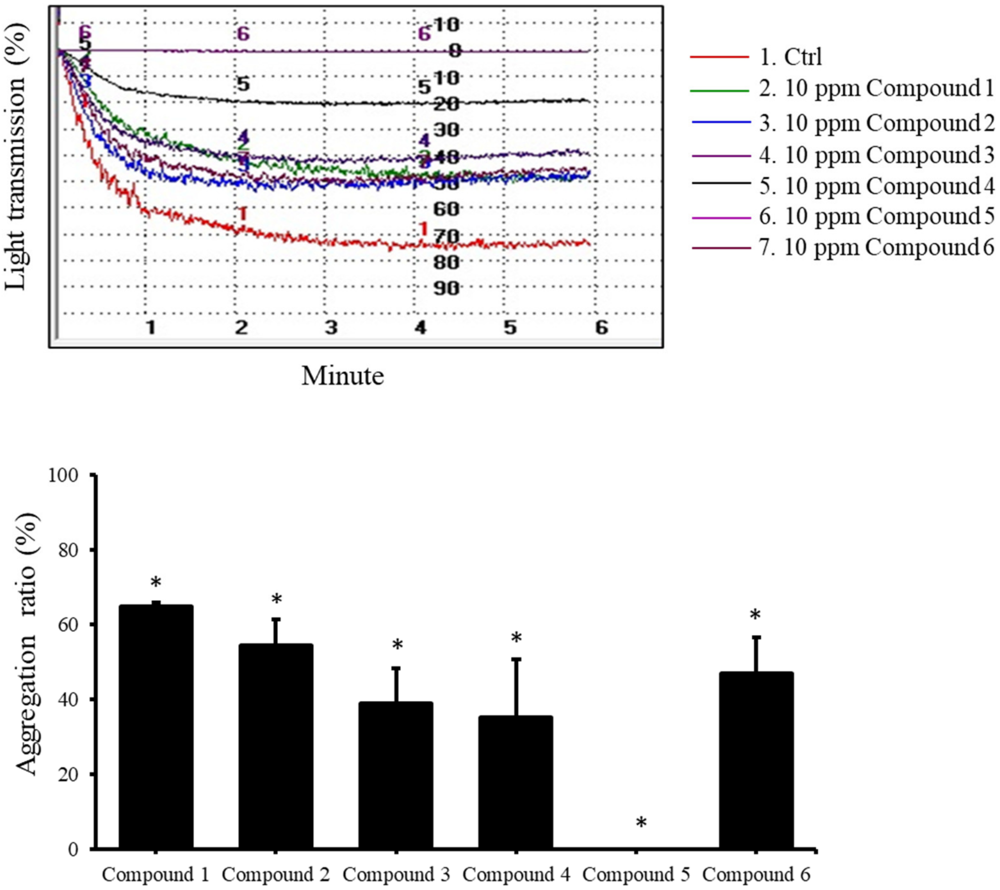

Figure S1. Effects of coumarin derivatives on collagen–induced platelet aggregation. Human PRP was treated with DMSO (control) or 10 ppm of coumarin derivates for 30 min, followed by addition of 5 μg/mL collagen, and aggregation was analyzed using an aggregometer for 6 minutes. The data are presented as the aggregation ratio relative to control group treated with collagen only (n = 3). *$p < 0.05$ compared with the control group treated with collagen only.

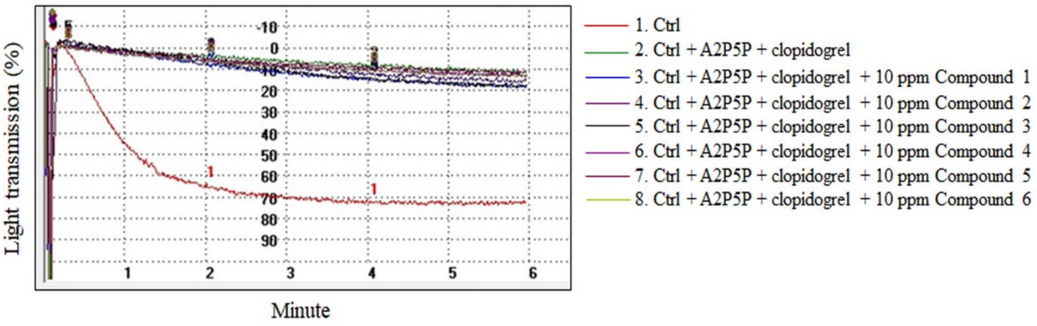

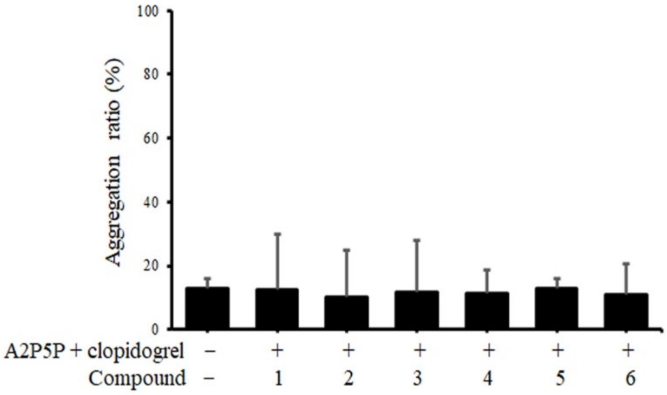

Figure S2. Effects of the ADP receptor antagonist on the inhibition of platelet aggregation by coumarin derivatives. Human PRP was treated with 10 ppm of coumarin derivates, 10 μM ADP alone or in the presence both of 1mM A2P5P and 1 mM clopidogrel. Aggregation was analyzed using an aggregometer for 6 minutes. The data are presented as the aggregation ratio relative to control group treated with ADP only (n = 3).

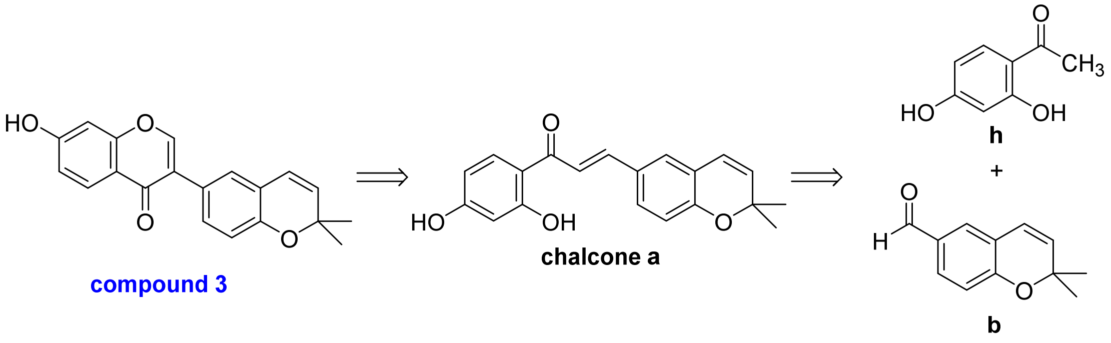

Scheme 1

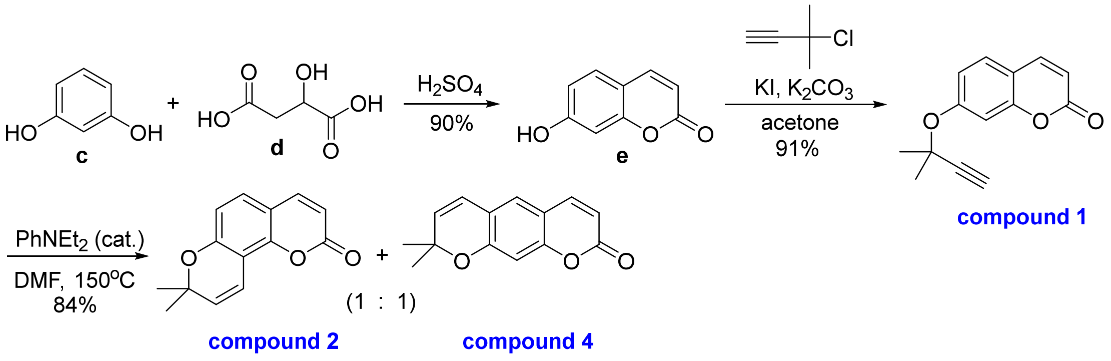

Scheme 2

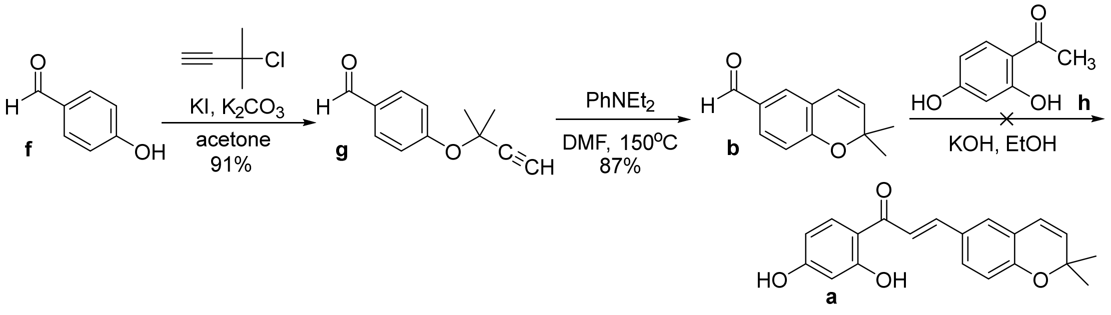

Scheme 3

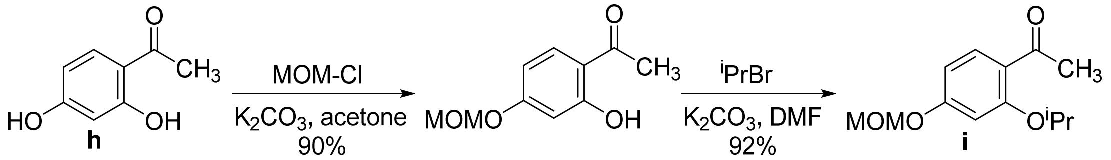

Scheme 4

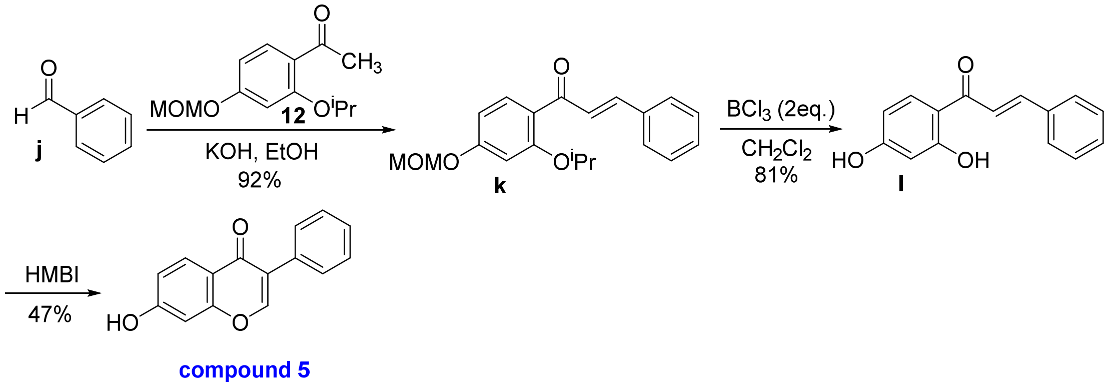

Scheme 5

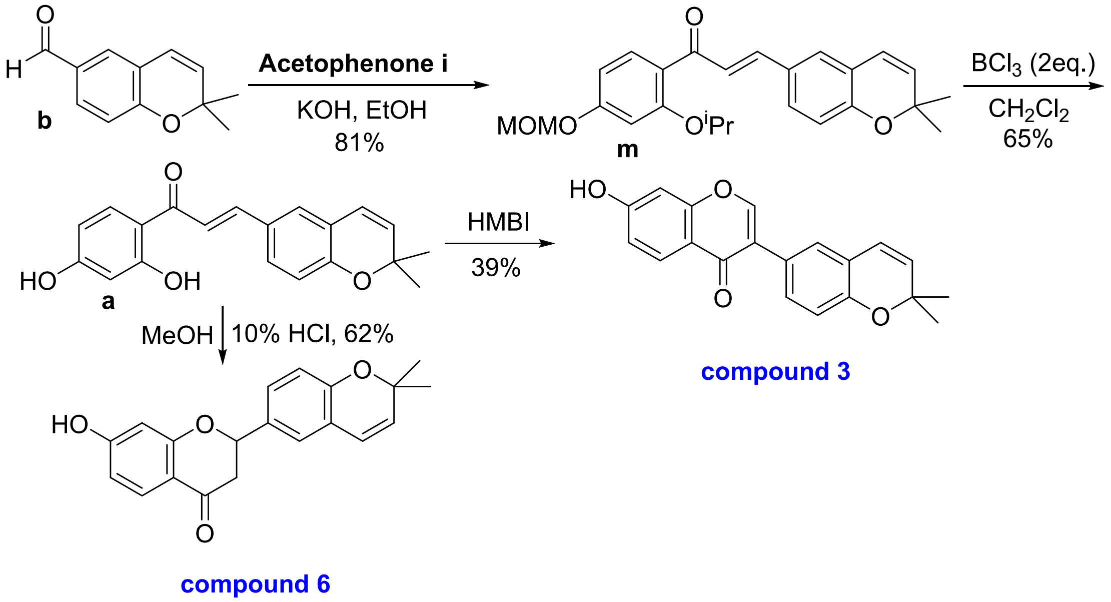

Scheme 6

Figure S3. Chemistry.

Using corylin **1** (**compound 3**) as our target product, there were several different synthetic methods to accomplish this synthesis. We used oxidative rearrangement of chalcone **a** to obtain the target product, and chalcone **a** was obtained via aldol condensation with acetophenone **h** and chromene **b** (Scheme 1). In the initial step, we used compound **e** as the starting material to establish the reaction conditions suitable for obtaining chromene **b**. Resorcinol **c** was reacted with 2-hydroxysuccinic acid **d** to yield coumarin 7-hydroxy-2H-chromene-2-one **7** with 90% yield. Compound **e** was reacted in 3-chloro-3-methylbut-1- yne, KI, K_2CO_3 after refluxing in acetone for 45 hours, and 91% of **compound 1** was obtained via extraction and purification. In the presence of a catalyst amount of $PhNEt_2$ and solvent DMF, a high-temperature cyclization reaction was performed at 150 °C for 10 hours. After extraction and purification, 84% of **compound 2** and **compound 4** were obtained (ratio 1:1). The mixture was purified through silica gel to obtain **compound 2** and **compound 4** with 42% yield (Scheme 2).

We used the same reaction conditions to convert aldehyde **f** to compound **b**. The yields of the two steps were 91% and 87%, respectively. We performed aldol condensation on compounds **b** and 2,4-dihydroxy acetophenone **h** under alkaline conditions, but the expected product was not obtained as a result of the reaction (Scheme 3). It is possible that intramolecular hydrogen bonds in acetophenone **h** prevent aldol reactions. Therefore, *O*-isoproxyl, *P*-MOM, and acetophenones **i** were used as the starting materials for alternative synthesis. The preparations of *O*-isoproxyl, *P*-MOM, and acetophenones **i** were straightforward. Acetophenone **h** was protected with MOMCl at the para-OH, and the ortho-OH was protected as -O^{i}Pr with isopropyl bromide, and approximately 83% of product **i** was obtained (Scheme 4).

The aldol reaction between acetophenone **i** and benzaldehyde **j** was performed to obtain chalcone **k** with a yield of 92%. Appropriate conditions for deprotection and oxidative rearrangement were then tested with compound **k**. The *O*-isopropyl ether and *O*-MOM ether were removed with BCl_3 (2 eq.) to afford chalcone **l** with 81% yield. We performed an oxidative rearrangement reaction with chalcone **l** and HMBI, which only 15% of the target product was obtained. We increased the purity of HMBI by altering the solvent and temperature of the reaction. Finally, oxidative rearrangement was performed at >95% purity of HMBI and chalcone **l** under refluxing MeOH to obtain 47% isolated **compound 5** (Scheme 5).

We used the same reaction conditions, aldol condensation of aldehyde **b** and acetophenone **i,** to yield chalcone **m** with a yield of 81%. Then, *O*-isopropyl ether and *O*-MOM ether were cleaved by 2 equivalents of BCl_3 to afford chalcone **a** in 65% yield. Finally, HMBI was added to perform oxidative rearrangement to obtain 39% of the target product **compound 3**. Alternatively, chalcone **a** was cyclized in 10% HCl to obtain **compound 6** with 62% yield (Scheme 6).
